# Modal Frequencies Associations with Musculoskeletal Components of Human Legs for Extracorporeal Bone Healing Assessment Based on a Vibration Analysis Approach

**DOI:** 10.3390/s22020670

**Published:** 2022-01-16

**Authors:** Benjamin Steven Vien, Wing Kong Chiu, Matthias Russ, Mark Fitzgerald

**Affiliations:** 1 Department of Mechanical and Aerospace Engineering, Monash University, Wellington Road, Clayton 3800, Australia; wing.kong.chiu@monash.edu; 2 The Alfred Hospital, 55 Commercial Road, Melbourne 3004, Australia; matthias.russ@russorthopaedics.com.au (M.R.); m.fitzgerald@alfred.org.au (M.F.); 3 National Trauma Research Institute, 89 Commercial Road, Melbourne 3004, Australia

**Keywords:** vibration analysis, fracture healing, bone assessment, human health monitoring, biomechanics, medical device, modal frequency

## Abstract

Reliable and quantitative assessments of bone quality and fracture healing prompt well-optimised patient healthcare management and earlier surgical intervention prior to complications of nonunion and malunion. This study presents a clinical investigation on modal frequencies associations with musculoskeletal components of human legs by using a prototype device based on a vibration analysis method. The findings indicated that the first out-of-plane and coupled modes in the frequency range from 60 to 110 Hz are associated with the femur length, suggesting these modes are suitable quantitative measures for bone evaluation. Furthermore, higher-order modes are shown to be associated with the muscle and fat mass of the leg. In addition, mathematical models are formulated via a stepwise regression approach to determine the modal frequencies using the measured leg components as variables. The optimal models of the first modes consist of only femur length as the independent variable and explain approximately 43% of the variation of the modal frequencies. The subsequent findings provide insights for further development on utilising vibration-based methods for practical bone and fracture healing monitoring.

## 1. Introduction

Bone fracture healing is a complex multifactorial process of restoring its biological function and mechanical properties [1,2,3]. Normally, the bone healing process is divided into three overlapping stages: inflammatory, bone reparative and bone remodelling [3]. The first stage immediately occurs with an inflammatory response in releasing cytokines, growth factors and prostaglandins and the formation of haematoma (blood clot) due to blood vessel rupture inside and surrounding the fracture site. The fracture haematoma becomes organised then forms a matrix for bone formation and primary callus. In the bone reparative stage, the soft callus holds the ends of the fractured bone, although cannot support sufficient weight-bearing, and converts into hard callus. Once the fracture ends are bridged by a hard callus (bony bridge), the callus size decreases, and the bone then remodels and restore the bone structure near to normal functionality which can take several years.

There is supporting evidence that well-regulated mechanical stimuli can strongly mediate the quality of healing at different fracture healing stages [3,4,5]. Initially, a fractured bone must be well supported to allow callus formation; however, prolonged inactivity or insufficient stress suppresses bone formation, hence delaying healing. Throughout the decades, radiographic techniques still remain as the conventional clinical diagnostic method, which involves visually interpreting planar radiographic images for features associated with fracture healing. Despite the existence of numerous different clinical and radiologic criteria used to assess healing, previous literature has reported that radiographic assessments are qualitative with a high level of uncertainty in recovery time; in the order of months, and radiographic features have poor correlations with bone strength and stiffness [6,7,8,9,10]. Furthermore, there is a lack of consensus among orthopaedic surgeons on the assessment and definition of fracture healing [11,12,13,14]. Thereby, there is a need for a reliable evaluation of fracture healing in both medical and research communities to improve orthopaedic diagnostics with the possibility to minimise radiation exposure and prompt early intervention. The ability to quantify healing based on the bone intrinsic properties allows real-time monitoring and prognostic capability, enabling data-rich comparative studies and, therefore, transforming our current practices towards a smarter trauma patient healthcare system.

Recent literature reviews [11,12,15,16] explored both qualitative and quantitative techniques to monitor fracture healing, which includes direct and indirect static measurements, ultrasound, acoustic and vibration measurements; refer to Table 1 for comparison. The methods that are implemented in clinical practices are primarily radiographic imaging techniques. Computed tomography scan (CT) is a superior radiographic imaging method for assessing fracture healing that provides quantitative measures of callus volume and bone mineral density earlier than conventional techniques. However, CT scan can be costly, has high radiation dosage, is not as widely available and the presence of fixations hinders its accuracy. In addition, bone scintigraphy has a very high sensitivity for the detection of bone inflammation and infection, although this qualitative technique requires the internal administration of radioactive substances a few hours before imaging.

There is significant research interest in employing mechanical properties assessment for fracture healing as an alternative non-radiative and quantitative approach with the capability of earlier detection and portability. In particular, bone stiffness is recognised as an essential parameter to evaluate the stage of healing as biological and physical regenerations of the bone changes its elasticity, which strongly correlates with the bone strength during callus formation. There are significant elastic modulus changes in the fracture site of at least five orders of magnitude to approximately 20 GPa for a fully healed bone [17,18,19]. The change in stiffness is most apparent during the early stages of healing as it has been reported that the stiffness increases exponentially during the consolidation phase. Some studies proposed a conservative estimate of a 25% bending stiffness recovery to be considered sufficiently healed [20,21]. Although direct measurements of deflection are the most accurate method in assessing stiffness, in some cases, it is considered destructive and not appropriate in the early weeks of fracture healing as newly formed calluses are susceptible to loading. Indirect biomechanical methods consist of attaching strain gauge onto the fixation columns and have shown early detection of fracture healing [20], although the method measures the change in stress in the fixation as the fracture heals and relies on the use of the same fixation system. Currently, both direct and indirect biomechanical techniques have mainly been validated for external fixations in the laboratory and in vitro settings. Recently, quantitative ultrasound and guided wave propagation were proposed as potential methodologies that utilise wave signal measurements on assessing healing fracture [22,23]. These methods rely on the appearance of wave modes and the change in wave velocity, which is caused by the difference in material properties of callus and cortices bone. However, the wave energy significantly dissipates into and damped by surrounding soft tissues and bone marrow, producing inaccurate results.

Vibration analysis methods, such as resonant frequency analysis (RFA), are non-invasive, non-radiative, painless alternative fracture healing assessments. These methods are based on engineering principles that the bone resonant frequencies have a direct correlation with its structure and stiffness, which are often detected by using accelerometers [24,25,26,27,28]. Early in-vivo studies [24,25] demonstrated the first resonant frequency; primarily a bending mode, as a measurand for monitoring fracture healing due to its sensitivity to callus formation. Benirschke et al. [26] has shown a strong correlation between bone healing and its stiffness when the elastic modulus has reached at least 5% of the intact bone. Furthermore, there are appearances of higher frequency waveforms in the response of a fully healed bone than of a fractured bone [29]. However, studies have shown significant variability in using RFA to evaluate healing due to mode coupling effects [25,30,31,32]. Many previous studies relied on instrumented impactors to facilitate the estimation of modal parameters in the bone frequency responses, which have been reported in some cases to be challenging to reliably measure [25,26,33]. A well-known challenge for vibration-based bone assessment is the damping effects of soft tissue. Tsuchikane et al. [34] has reported that surrounding soft tissue and joints increase the apparent weight of the long bones, resulting in a dampened frequency response. The presence of soft tissue is shown to dampen the magnitude of the vibrational response as well as greatly masks higher frequency modes [31,35,36,37,38]. Therefore, in formulating a vibration-based assessment tool, a significant amount of research and data is essential to examine and, feasibly, dissociate the adverse effects arising from soft tissue and other body parts.

Recently, there is a re-emergence of interest in utilising vibration analysis methods for quantitative bone assessment [31,33,35,39,40,41,42,43,44,45,46,47,48,49]. Previous works by Chiu et.al [35,40] have employed a two-sensor strategy to identify different modes without the input response of instrumented impactors and have shown that the torsional mode is more sensitive to the recovery of the osteotomised region with minimal influence by the stiffness of the fixation system and mass-damping effect of soft tissue. Chiu et al. [41] also indicated that there is no significant difference between transverse or oblique fracture orientations after 1% recovery of the healed bone stiffness. Studies have proposed indices based on the frequency response of a healing bone to quantify the stages of healing of which its temporal profile resembles that of a recovering bone stiffness [13,20,44]. Recent works [44,49] have indicated that vibration analysis methods are most applicable in the early stages of healing (i.e., from zero to seven weeks).

Extensive and highly significant works by Mattei et al. [44,45,46,47] demonstrated the validity of vibration analysis methods for assessing the healing of fracture treated with an external fixator. Their recent clinical case study [44] has shown a significant sensitivity in a low resonant frequency in the vicinity of 60–100 Hz, with an equivalent stiffness change of approximately 50% relative to a baseline taken at the end of the limb lengthening procedure. Furthermore, Mattei et al. also observed different rates of change in resonant frequencies which are ascribed to the complex healing processes including those other physiological factors, such as muscle tone, than that of the callus stiffening. Their findings correspond well with previous studies, which have shown that the healing rate in areas corresponding to tissue, cartilage and callus formations are different, and as well as has a positive correlation with strains [3,4,5].

Nevertheless, vibration analysis methods employed for fracture bone healing assessment are rare as clinical studies are [24,44,45,46,47] scarce and, thereby, currently are not widely accepted by clinicians. Furthermore, the lack of large-scale validation, dataset and clinical reports investigating the reliability of vibration analysis methods presents a major impediment to progress into clinical and research practices as well as advances in human health monitoring.

The aim of this study is to experimentally determine the modes and their associations with human bone and measured body (a majority of leg components) components of healthy participants via a clinical trial in facilitating the development of quantitative healing assessment using vibration-based methods. A prototype design based on the patented method and system by Chiu, Russ and Fitzgerald [50] is employed to demonstrate and evaluate the application of the vibration-based method. Lastly, mathematical models are formulated to relate the modal frequencies and their associated body components. Furthermore, the proposed strategy, including the effectiveness of the prototype device, is also discussed.

## 2. Materials and Methods

### 2.1. Prototype Design

**Figure 1:**
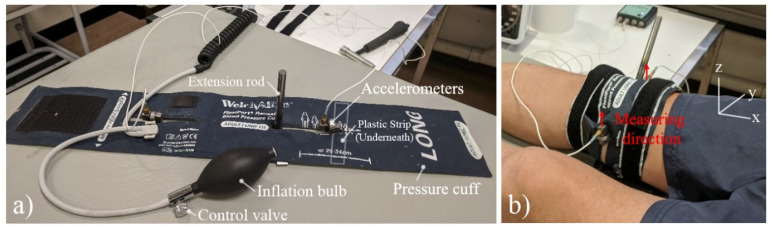
(**a**) Labelled prototype device and (**b**) installed prototype device on participant’s knee.

The patented prototype device (shown in Figure 1) is a radiation-free, non-invasive and painless monitoring apparatus that utilises vibration-based methods to quantitatively assess bone stiffness, thereby fracture healing. The benefits of the device include a portable rapid assessment of fracture healing without exposure to radiation, an objective approach based on the intrinsic properties of the bone and suitability for various patient sizes.

A reusable blood pressure cuff is modified with two slots located at the centre of the cuff, allowing adjustments of accelerometers locations for different leg sizes. The cuff is connected to a port fitting aneroid sphygmomanometers and with a manual inflation bulb and control valve. Two Brüel and Kjær Miniature Constant Current Line Drive (CCLD) Accelerometers Type 4507 were used with side-mounted attached to mounting clip (upper limiting frequency of 3000 Hz), which were bonded to a flexible rectangular plastic strip.

The participants were in the supine position (lying horizontally and facing up) to minimise the effects of muscle, as muscle contraction and relaxation can significantly change its elastic modulus at least an order of magnitude [51], and maintain the same boundary conditions for repeatability. The prototype device was installed on the participant’s distal femur (slightly above the knee) and the accelerometers were aligned and secured at the medial and lateral epicondyles, where the femur bone has the least coverage of muscle, to maximise the modal response of bone whilst minimise the damping effects arising from soft tissues. The accelerometers measure in the direction tangential to the femur; the anterior-posterior direction (positive z-direction), as indicated in Figure 1b. The pressure cuff was gradually inflated to 180 mmHg to ensure sufficient stability throughout the procedure and the pressure cuff bled on average to 170.9 mmHg with a standard deviation of 3.5 mmHg. In the preliminary investigation on the pressure cuff, the frequency responses with pressure greater than 120 mmHg were sufficiently stable. An extension rod was also included and lightly tapped using an impact hammer strike to induce a torsional response. Although this two-sensor strategy requires no knowledge of the input signal, for consistency, Brüel and Kjær Impact Hammer type 8206 with an aluminium tip, which has a well-sufficient frequency spectrum of impact force (approximately cut-off 10 kHz), was used for this study.

Data was acquired through a Two-channel Brüel and Kjær Photon+ and the signal responses were processed using RT Pro Photon software. The software analysis parameters were sampled at 4096 Hz (time step of 244.1 μs) with a frequency resolution of 1 Hz. A test consists of 10-spectrum linear averaging to minimise random noise and randomly excited non-linearity from the cross-spectrum of the signal pair. To ensure reliable signals, the trigger is automatically armed with a trigger threshold of 0.1 V with a bi-polar slope of 1% level. Once the test was completed, the pressure cuff was then deflated and the device was removed. The duration of the test took 30 s. This process was repeated 10 times (10 tests) on each leg per participant. Prior investigation on the prototype device indicated that at least 3 tests are required to reveal almost all (>80%) modes.

The least-squares rational function estimation-based approach via MATLAB’s System Identification Toolbox™ [52] was used as a curving fitting approach to estimate experimental modal parameters from the cross-spectra. The estimated modes satisfy the stabilisation criteria with a tolerance of 1% for the modal frequency and 5% for the damping coefficient. Coherence value exceeding 0.9 is considered high-quality measurements and phase values of [0, ¼π), [¼π, ¾π] and (¾π, π] are considered in-phase, coupled and out-of-phase (OOP) modes, respectively.

### 2.2. Clinical Trial

The clinical trial, ‘Pre-market pilot study of a non-invasive and painless device to measure fracture healing stage’, was conducted in Human Laboratory in Department of Physiology, Monash University Clayton and approved by Monash University Human Research Ethics Committee (MUHREC); MUHREC Approval Project Number 2020-23688-50577. A total of 20 (12 males and 8 females) healthy participants, aged between 22–59 years old (mean age of 27.2 years old and standard deviation of 7.96) with no current leg fracture and able to walk unassisted, volunteered in this clinical study and a total of 40 legs (400 tests) were investigated. Upon completion of the trial, the participants were given a personalised body composition measurement scan result and a Woolworth/Coles $20 gift card.

The clinical trial procedure begins with a preliminary acquisition of body variable measurements followed by installation and testing of the prototype device, which is summarised in Table 2.

The InBody 770 Scan body composition analyser was used to non-invasively measure the participant’s body compositions, including segmental analysis of individual legs, which were comparable to those acquired via dual-energy X-ray absorptiometry (DEXA) scan [53,54]. In contrast to the conventional bioelectrical impedance analysis, the InBody fat-free mass measurements have shown a 98.4% of correlation with those measured using DEXA. Succinctly, InBody device is an 8-point tactile electrodes system that uses direct segmental multi-frequency bioelectrical impedance analysis for each body segment (4 limbs and trunk) by applying alternating low-level electrical currents through the water in the body. The InBody device utilises 30 impedance measurements by using different 6 frequencies between 1 kHz–1 MHz to extract over 50 parameters relating to body compositions within minutes [55]. The participants were on an empty stomach and bladder, wore loose comfortable clothing without any metal, removed their socks and cleaned their hands and feet prior to the body scan. During the body scan analysis, the participant maintains an upright test posture (arms not touching the side of the body and thighs are not touching) while standing on the foot electrodes and holding the hand electrodes and avoiding contact and talking. The height of the participants was measured using the Height Measuring system SECA 264. Additional manual measurements include lower and upper thigh girth measurements at 5 and 15 cm, respectively, proximal to the superior pole of the patella (knee cap) and true leg length measurement (from the anterior superior iliac spine to medial malleolus) [56]. Previous works indicated strong correlations between height and femur length [57] as well as strong relationships between tibia and femur length with a tibia-femur ratio of 0.8 and a standard deviation of 0.03 [58]. Thereby, in this study, any correlation with leg length is considered as so with the femur length and in formulating a mathematical method, femur length is assumed to be proportional to leg length.

### 2.3. Data Preprocessing

#### 2.3.1. Descriptive Statistics of Participants

The InBody scan and manual measurements resulted in over 60 variables and were pre-processed to retain relevant body components. First, components not associated with the leg or considered irrelevant (i.e., trunk, arms, etc.) were disregarded. A dimensional reduction technique using Spearman’s rank correlation coefficient was then employed to determine monotonic nonlinear associations between variables, which includes those that were not necessarily a linear relationship. Specifically, pairwise correlations between each pair were analysed and those with strong correlation coefficients beyond 0.9 and very high statistical significance (p<0.001) were removed. This reduced the body components to height (h), lower and upper thigh girths ($t_{L}$ and $t_{U}$, respectively), leg length (l), weight (w), segmental lean ($s_{L}$) and fat leg ($s_{F}$) masses; refer to Table 3.

During the preliminary statistical analysis, there were significant differences between male and female body components; specifically, height, lower thigh girth, weight and segmental lean and fat leg masses. Upon visual inspection, the data suggested identical normal-like distributions in both groups and thereby, an assumption was made that the analysis was treated as a family of normal distributions. Firstly, we suspected the presence of outliers in the dataset may cause the test to reject normality and so the outlier test using the interquartile range method was performed to identify and remove extremities. Next, two-sample F-tests were conducted to validate the assumption of the null hypothesis of equal variations in both gender groups and suggested unanimously failed to reject the hypothesis with a significance level of α=0.05. Therefore, the data from both groups were pooled and then tested for normality using the Lilliefors (modified Kolmogorov–Smirnov) test. For all components, the test statistics did not exceed the critical value, indicating that the components can be interpreted as normally distributed. The above preliminary analysis supported the assumption of the normal distribution type of the investigated body components and so the study proceeded in analysing the associations among body components and modal parameters with both gender data pooled. In this study, p<0.05 is considered statistically significant and those of high significance (p<0.01) as well as of slightly moderate significance (0.05≤p<0.10) are also discussed.

#### 2.3.2. Clustering of Modal Parameters

**Figure 2:**
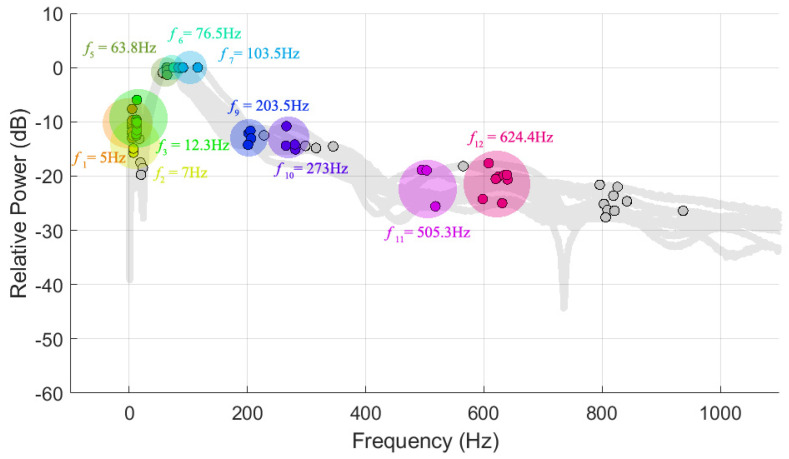
Schematic of the mode clusters using DBSCAN projected in two-dimensional space of a leg frequency response.

The modal and system parameters extracted were modal frequency, damping ratio, relative power, magnitude and phase of the cross-spectrum and coherence. The challenge was to accurately categorise and distinguish modes as some modes may vary in frequency due to their dependences on body components. In order to determine the associations among the modal parameters and the body components, a clustering algorithm using Density-based spatial clustering of applications with noise (DBSCAN) was performed to discover distinct groups and as well as noise in multi-dimensional data [59]; refer to Figure 2. The DSCAN algorithm works on the assumption that clusters are dense regions separated by regions of lower density and uses the principle of searching for neighbourhoods of data points that exceed a certain density threshold. The DBSCAN algorithm initiates with an arbitrary point and retrieves all points directly density-reachable (within the radius, ε, of a neighbourhood) and satisfies the minimum of points (MinPts) required to be a neighbourhood. A density-based cluster is formed by merging overlapping neighbours, where points in the cluster are density-reachable from their surrounding points. If the arbitrary point is on the border of a cluster, thereby no points are density-reachable from the arbitrary point and then the algorithm proceeds to the next point of the dataset and the procedure is repeated until all points are visited. Points that are not reachable with respect to ε and MinPts are considered not part of a cluster and hence are identified as outliers. In this study, a DBSCAN with Euclidean distance metric was performed on the 10 tests for each leg with a MinPts of 3 and the average threshold of ε of 3 and 16 for modal frequencies in the range of 0–40 and 40–1000 Hz, respectively. It should be noted, ε values were first estimated using k-nearest neighbour search and adjusted, though, significantly for noisy cross-spectra. Afterwards, the average values of the modal parameters for each cluster were calculated and assigned into 12 distinct mode groups, where modes ‘1, 2, …, N’ are denoted as $\Omega_{1,2,\ldots \;,N}$ and the corresponding average modal frequencies as $f_{1,2,\;\ldots ,N}$. It should be noted that not all leg frequency responses have 12 distinct modes.

In addition, the appearance of each of the 12 modes was also investigated to determine any dependency of body components. The modal frequencies were converted to a nominal scale to observation (*O*) and no observation (*N*) of the modal frequency, and then Wilcoxon rank sum test was performed to test against the two nominal groups for each body component. The effectiveness of the proposed strategy, inclusive of the prototype device, was then evaluated and discussed.

### 2.4. Model Formulation Using Stepwise Regression

In vibration theory, the modal frequency response depends on the geometry and composition of the structure. For a linear elastic, uniform and cantilever beam, the natural transverse and torsional frequency [28] is:

(1) $$f_{transverse}=\frac{\alpha }{2\pi }\sqrt{\frac{EI}{pAL^{4}}},\;f_{torsional}=\frac{\alpha }{2}\sqrt{\frac{GK}{pJL^{2}}}$$

where E is Young’s Modulus of elasticity, G is the shear modulus, I is the area moment of inertia, J is the polar moment of inertia about the axis of rotation, p is density L is the total length of the beam, A is the cross-sectional area, K is the geometric function of the cross-sectional area and α is the constant (equivalent to modal eigenvalue) depending on the type and order of the vibrational mode and boundary conditions. An approach is to construct a simplified model (Equation (1)) of which the system behaves (i.e., implementations of spring and damping systems in modelling human leg as a uniform beam vibration) [27,30,32]. This predetermined equation approach may not be accurate as it requires significant simplification and assumption of the variables, such as uniform thickness and cross-section and boundary conditions, since some variables (i.e., J, I, K and A) are difficult to directly measure or estimate. Furthermore, it is also known that body components are also dependent on each other (i.e., cortical cross-sectional bone area increases with increasing femur length) [57,60].

An alternative approach is that the frequency formula is viewed as a multivariable power-law relationship which can be formulated using the measured variables (body components), instead of the prescribed independent variables in the previous approach.

(2) $$f=\alpha_{0}\sqrt{\overset{N}{\underset{i=1}{\prod }}Variables_{i}^{n_{i}}}$$

The measured and dependent variables are transformed using logarithmic transformation, thereby linearising the expression to a linear form.

(3) $$\log\left(f\right)=n_{0}+\overset{N}{\underset{i=1}{\sum }}n_{i}\log\left(Variables_{i}\right)$$

We proceed with further simplifications and so,

(4) $$F\left(V_{1},V_{2},\;\ldots ,V_{N}\right)=n_{0}+\overset{N}{\underset{i=1}{\sum }}n_{i}V_{i}$$

where $V_{i}=\log\left(Variable_{i}\right)$, F=log(f) and the square root is absorbed in $n_{i}$ and for mathematical consistency, the logarithm of the coefficient $\alpha_{0}$ is expressed as $n_{0}$. In this form, the strongly associated variables have a coefficient $\left|n_{i}\right|\gg 0$.

For the development of a baseline model, for all modal frequencies, all measured body components; height, leg length, lower and upper thigh girth, segmental lean and fat leg masses were considered as the measured variables. However, leg length was replaced by femur length, $l_{F}$, (equating to 5/9 of leg length) instead. The transformed (logarithmised) measured variables are denoted with uppercase (i.e., $T_{u}=\log\left(t_{u}\right)$, $L_{f}=\log\;(l_{f})$, etc.). Therefore, the logarithmised version of the above equation is simply expressed as,

$$F_{j}\left(\tilde{V}\right)=\tilde{n}.\tilde{V}$$

where

(5) $$\tilde{V}={\left[1\;H\;L_{F}\;T_{L}\;T_{U}\;S_{L}\;S_{F}\right]}^{T}\;\tilde{n}=\left[n_{0}\;n_{H}\;n_{L_{F}}\;n_{T_{L}}\;n_{T_{U}}\;n_{S_{L}}\;n_{S_{F}}\right]$$

However, it is anticipated that some of these measured variables are dependent on each other. A stepwise multiple linear regression model (*stepwiselm* in-built MATLAB function) was implemented to sequentially process for terms to be added or removed in determining the optimal model. With a level of significance α<0.05, the criteria are based on the p-value of the F-statistic for each coefficient of the terms in the linear model and of the F-test of the regression model on whether the model fits than a degenerate model consisting of only a constant term. At each step, the *stepwiselm* function checks for linear dependencies among the terms in the model and if a term is linearly dependent, the function removes the redundant term. The regression models were discussed and assessed based on their standardised residuals, adjusted coefficient of determination ($R_{adj}^{2})$, standard error (SE) of the coefficients and coefficient of variation (CV).

## 3. Results

### 3.1. Modal Parameters

The 12 modes from 0–800 Hz were identified, which were primarily OOP and coupled modes and their associated coherence suggested high-quality measurements; refer to Table 4. The dominant modes are $\Omega_{5–7}$ depicted by their associated relative power and amplitude and in the vicinity of 50–110 Hz. Higher modal frequencies beyond 250 Hz, $\Omega_{10–12}$, are significantly weaker modes (<−14 dB). OOP modes are $\Omega_{1,3–6}$ whereas the remaining modes are identified as coupled modes. The variations (difference between the maximum and minimum frequency) of the modes are mostly larger for higher modal frequencies. The modes with high damping coefficients of approximately 0.2 are $\Omega_{1,5–7}$ and the low damping coefficients are of high modal frequency $\Omega_{9–12}$. On average, eight modes were identified in leg frequency response, where $\Omega_{1–3}$ were recorded the most with approximately 87%, $\Omega_{4}$ was recorded the least with 18%, $\Omega_{6,7}$ were recorded approximately 47% and $\Omega_{8–12}$ were recorded approximately 63% of 40 tests.

### 3.2. Association Analysis

**Figure 3:**
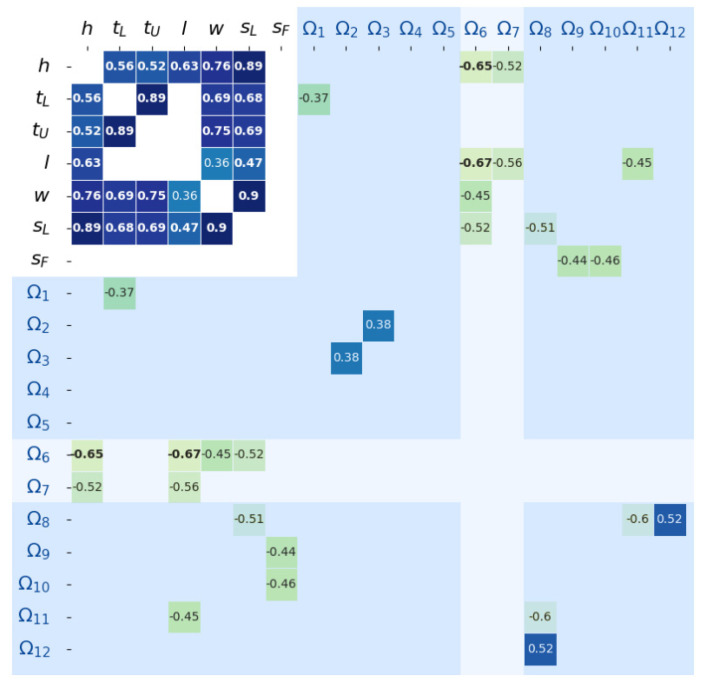
Spearman’s correlation heat map showing correlation coefficients of significances p<0.05 and (bolded) *p* < 0.01.

The next investigation proceeded in discovering associations among the 12 modes and body components, and those with high significance are indicated with their associated correlation coefficients in Figure 3.

Most body components have a moderate to strong (0.4~0.9 coefficients) correlation with high significances, in particular the correlation between weight and segmental lean leg mass, between height and segmental lean leg mass and between lower thigh and upper thigh girths. Weight has a weak positive correlation with leg length. There is no correlation with statistical significance between segmental fat leg mass and the other body components and upon further investigation, there is a weak correlation (coefficient of 0.31) between weight and segmental fat leg mass with a significance of p≅0.05.

### 3.3. Modal Frequency Association with Body Components

Referring to Figure 3, the modal frequencies and their correlations of statistical significance with the body components are stated below.

- $\Omega_{1}$ has a weak negative (coefficient of −0.37) correlation with the lower thigh girth.
- $\Omega_{2–5}$ have no correlation with the body components. There is a weak (coefficient of 0.38) correlation between $\Omega_{2}$ and $\Omega_{3}$.
- $\Omega_{6}$ has a moderate negative correlation (very high statistical significance of p~0.001) with height and leg length. Furthermore, $\Omega_{6}$ also have fair negative correlations with weight and segmental lean leg mass, which is likely due to their correlation with leg length. Thereby, $\Omega_{6}$ is considered the first OOP mode associated with the femur length.
- $\Omega_{7}$ also has moderate negative correlations with height and leg length. $\Omega_{7}$ is considered the first coupled mode associated with the femur length.
- $\Omega_{8}$ has a moderate negative correlation with segmental lean leg mass. $\Omega_{8}$ also has negative and positive correlations with $\Omega_{11}$ and $\Omega_{12}$, respectively.
- $\Omega_{9}$ and $\Omega_{10}$ have a moderate negative correlation with segmental fat leg mass.
- $\Omega_{11}$ has a moderate negative correlation with leg length.
- Though there is no correlation with statistical significance between $\Omega_{12}$ and the body components, a further investigation found that $\Omega_{12}$ has a weak negative correlation (coefficient of −0.33) with segmental fat leg mass with significance p≅0.07.

Due to $\Omega_{1–5}$ having weak and no correlations with the body components, these low-frequency modes are anticipated to be associated with the prototype device and its components. It is possible that there are associations with other not-measured and/or low-variation body components, such as skin [31]. The particular interest of this investigation is the modal frequencies within the range of 60–400 Hz, largely due to their association with the musculoskeletal components of the leg. $\Omega_{6,7}$ have strong correlations with leg length and, thereby, with femur length, which suggest these modes serve as primary measurands for bone healing assessment. Furthermore, $\Omega_{8–10}$, as well as the higher-order, coupled modes $\Omega_{11,12}$, are quantities that describe the lean and fat masses of the leg and despite their relatively weaker magnitude, these modes are potential supplementary for healing evaluation.

### 3.4. Dependency of Modes Appearances

The Wilcoxon rank sum test suggested statistically significant differences between the *O* and *N* groups for $\Omega_{7,8,10}$, refer to Table 5, thereby suggesting the appearance of these modes are dependent on body components. The appearance of $\Omega_{8}$ occurs when the body components are higher in value, in particular weight and segmental lean leg mass with high significance (p<0.003). The appearance of $\Omega_{10}$ occurs when weight and segmental lean leg mass are lower in value. Similarly, the appearance of $\Omega_{7}$ occurs when weight and segmental lean leg mass are lower in value; however, with slightly moderate significance. This indicates that $\Omega_{7,10}$ are masked (damped) by increasing muscle mass and weight.

### 3.5. Model of Modal Frequencies Associated with Leg Components

The stepwise regressions procedure showed only $\Omega_{6–11}$ models satisfy the criteria, refer to Table 6. Each optimal model has only one independent measured variable; the independent measured variable for $\Omega_{6,7,11}$ models is $l_{F}$, for $\Omega_{8}$ model is $s_{L}$ and for $\Omega_{9,10}$ models is $s_{F}$. An additional investigation was conducted to investigate all combinations of measured variables and found that only these optimal models satisfied the criteria. Among the models, $\Omega_{6}$ and $\Omega_{7}$ models have high statistical significance and CV of $n_{L_{F}}$ and $n_{0}$ of approximately 27% and 14.5%, respectively. $\Omega_{8–10}$ models have a relatively low CV of $n_{0}$ of less than 6%; however, a relatively high CV of their variable coefficient of approximately 40% compared to those of $\Omega_{6,7}$ models. $\Omega_{11}$ model has a CV of $n_{0}$ of approximately 12.2% and the largest CV of $n_{L_{F}}$ of 42.2%.

**Figure 4:**
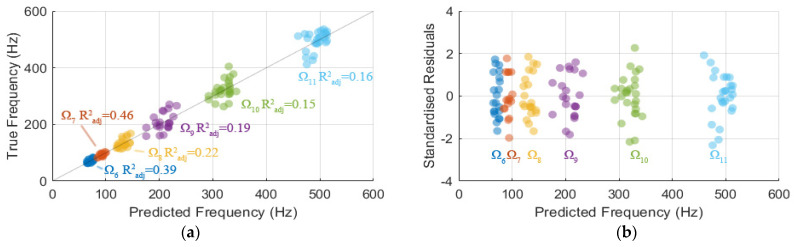
(**a**) Graph of predicted and true modal frequencies and (**b**) graph of standardised residuals against predicted frequency.

Referring to Figure 4, the variations in $f_{6}$ and $f_{7}$ are considerably explained (on average 43%) by their models with $l_{F}$ as the independent variable, whereas approximately 15–20% variations of $f_{8–11}$ are explained by their associated model with one independent variable ($s_{L},s_{F}$ and $l_{F}$, respectively).

There is no observation with a standardised residual outside of ±3, which indicates a region where the fitted model is considered a poor approximation. Furthermore, there is no clear indication of bias nor heteroscedasticity, hence the errors are considered normally, independently and identically distributed. Therefore, the models can be considered appropriate approximations for the modal frequencies.

## 4. Discussion

The results indicate $\Omega_{6,7}$ modal frequencies of 71.59 and 92.71 Hz, respectively, are associated with the femur length, as it is correlated with leg length [58], as mentioned previously. These modes are in the same vicinity of modal frequencies associated with fracture healing reported in previous clinical studies where long bones (tibia and femur) were treated with an external fixation [44,46]. It should be noted that the previous studies reported first bending modes, whereas in this study, $\Omega_{6,7}$ are the first OOP and coupled modes, respectively. Nevertheless, these first-order modes can be primarily utilised to construct assessment models for fracture healing. In a fixated fractured bone frequency response, there exist modes associated with the internal/external fixation, which may couple and mask modes associated with healing due to its rigidity [25]. Though, our prior investigations [35,40] demonstrated that OOP and torsional modes are minimally influenced by fixations. Further validations are necessary for future investigations in employing the extracorporeal device which should include measurable information on bone properties and different scenarios of fractured bones.

Modal frequencies ($\Omega_{8–10}$) associated with the segmental lean and fat mass of the leg are very similar to those identified as the frequencies of the patient’s thigh, which are 122, 191, and 321 Hz [46]. Among those modal frequencies, $\Omega_{8}$ is the most damped (average damping coefficient of 14%), which is in agreement with previous related works [45,46]. The association of lean and fat leg masses with $\Omega_{8}$ and $\Omega_{10}$, respectively, are also evident due to their likelihood of appearance with muscle leg mass. Furthermore, the vibration responses also indicate stronger peaks amplitude at low frequencies and weaker peaks amplitude at high frequencies with higher variations, which are similar observations to the studies reported by Mattei et al. [44,45,46].

Despite the strong dependences among body components in the study findings and previous literature [57,60], the simplicity of the optimal models (describes with one variable) suggests these OOP and coupled modes, in particular $\Omega_{6,7}$, are minimally influenced by other musculoskeletal components of the leg. This is in accordance with our previous findings [35,39,40] that torsional modes associated with fracture healing are not sensitive to the mass-damping effect of soft tissue. In the view of utilising these models, we postulate the parameter $n_{0}$ can be used for bone quality and healing assessment as it essentially relates to bone properties (i.e., stiffness, density, etc.). However, there are some variations of $n_{0}$ in the models which are anticipated due to variation of quality of femur bone among the participants. In this current work, the models are only presumably limited to healthy bone, specifically, of ‘young adults’ as the majority of participants are in the 20–30 years old age range. Through visual inspection, the $\Omega_{6,7}$ modal frequencies for age ≥30 years old (three participants) are in the lower bound of the models, which imply lower bone stiffness. Nonetheless, the number of samples in the dataset is insufficient to statistically validate any observation or correlation against age and bone properties. Prior investigations [44,49] have indicated that vibration analysis methods are most sensitive from the inflammatory stage to the bone reparative stage due to the significant change in rigidity (blood clot to hard callus) of the fracture site. However, the limitation of this current study also includes the frequency responses of unhealthy participants (with fractured bones) as they were not considered in this study. Thereby, the modes associated with fractured bones as well as the applicability of this strategy is currently not experimentally validated in the early stages of healing. The current findings, as well as the models, can be further refined with more samples, including those in different age groups since bone mechanical properties change with increasing age [60], and to investigate $n_{0}$ sensitivity for healing assessment inclusive of participants with fractured bone.

The current vibration-based strategy was able to moderately (on average 55%) acquire modal frequencies associated with the femur, lean and fat leg mass. The $\Omega_{7}$ appears for lower values of weight and segmental lean mass, and this is in correspondence with the clinical trial observations where participants with muscular thighs have more soft-tissue coverage over the medial and lateral epicondyles, resulting in some difficulty for sensor placement and adverse masking of the femur response. Improvements on the strategy should include reducing the number of tests for reliable modal frequency acquisition and other considerations for the prototype device (i.e., sensor placement to further accentuate the femur and other leg components responses). Furthermore, the current procedure requires a well-versed operator to perform the analysis and identify the modal frequencies using signal pre-processing techniques for vibration signals, as well as to locate the epicondyles for sensor placement. Nevertheless, the research project will include not only further validations on the practical use of the vibration-based method, but also a much-needed simplification for clinical use.

## 5. Conclusions

This study demonstrates a patented prototype device using a vibration analysis method to measure modes associated with musculoskeletal components of the leg. The work aimed to provide insight in formulating indicators to assess fracture healing and broaden the knowledge for vibration-based healing assessment. A clinical trial was conducted to experimentally extract, by using the prototype device, and analyse the leg vibrational response of healthy participants against measured body components. On average, 55% of the modal frequencies associated with the femur, lean and fat leg masses were acquired via the proposed strategy. The first OOP and coupled modal frequencies of 71.59 and 92.71 Hz, respectively, are associated with the femur length. In particular, the first OOP mode has a high significance of p<0.003 and thereby, are potential bone healing and/or quality measurands. The findings have shown higher-frequency and higher-order modes associated with muscle and fat mass of the leg, with average damping coefficients between 0.12 and 0.14. In addition, higher modal frequencies beyond 250 Hz are significantly weaker modes, which are lower than −14 dB in relative power. Furthermore, $\Omega_{6–11}$ models were formulated using the stepwise regression method in approximating the modal frequencies with their associated body variables for healthy subjects. The variations in the frequency of the first OOP and coupled modes are explained on average 43% by their optimal models, with femur length as the only independent variable. Future work is currently underway to further validate the employment of vibration analysis approaches in facilitating smarter human health monitoring and medical devices for fracture healing assessment.

## 6. Patents

Chiu, W.K., M. Russ, and M. Fitzgerald, Australian Patent No. 2019900018. Method and system for assessing the state of healing of a fractured long bone. 2019: Australia, IP Australia.

## Figures and Tables

**Table 1:** Comparison of different assessment methods of fracture healing.

	Conventional Radiography	Computed Tomography	Bone Scintigraphy	Direct Biomechanical Testing	Indirect Biomechanical Testing	Vibration Analysis Method	Quantitative Ultrasound/Guided Wave
**Strategy**	Imaging Measures	Imaging Measures	Imaging Measures	Mechanical Properties Testing	Mechanical Properties Testing	Mechanical Properties Testing	Mechanical Properties Testing
**Clinical practice**	Y	Y	Y	N	N	N	N
**Non-destructive/** **Non-invasive**	Y	Y	Y	N	Y	Y	Y
**Non-radiative**	N	N	N	Y	Y	Y	Y
**Qualitative/Quantitative**	Qualitative/ Relative quantitative	Quantitative	Qualitative	Quantitative	Quantitative	Quantitative	Quantitative
**Principle of Evaluation**	Relative bone mineral density	Callus volume and bone mineral density	Radioactivity in tissues	Deflection under certain load	Strain	Resonant frequency	Wave velocity and modes
**Earliest Stage of Healing Detection**	Reparative	Inflammatory	Inflammatory	Reparative	Inflammatory	Inflammatory	Inflammatory
**Other Limitations**	- Lower resolution contrast in comparison to other radiographic techniques.	- Costly and not widely available. - Challenging with the presence of fixations. - High radiation dosage for regular usage.	- Images are acquired a few hours after injection. - Unreliable with the presence of internal fixations	- May not be acceptable during the early stages of healing due to loading. - Not validated in internal fixation devices.	- Suitable for only patients with external fixation. - Different results for different fixation systems.	Surrounding soft tissue and joints significantly obscure the readings.	Surrounding soft tissue and joints significantly obscure the readings.

**Table 2:** Description of clinical trial procedure in each stage.

Preliminary Stage			Device Testing Stage	
InBody Scan	Manual Measurements		Installation of Device	Testing of Device
Participant maintains test posture on InBody Body Scan to obtain body parameters.	Participant stands upright to acquire thigh circumferences.	Participant lays (supine position) on medical examination bed to measure leg lengths.	Whilst the participant is in supine position, the device is installed on participant’s knee and inflated to 180 mmHg.	Extension rod is struck to obtain 10-spectrum averaging. The pressure cuff is then deflated and removed. This process is repeated 10 times for each leg.

**Table 3:** Descriptive statistics of the relevant body components.

	Height	Lower Thigh Girth	Upper Thigh Girth	Leg Length	Weight	Segmental Lean Leg Mass	Segmental Fat Leg Mass
**Units**	cm	cm	cm	cm	kg	kg	kg
**Mean**	173.32	42.36	50.30	93.48	71.62	8.49	2.69
**Minimum**	163.50	33.70	43.20	87.00	51.70	5.94	1.20
**Maximum**	189.20	53.00	64.00	104.00	117.20	11.89	6.10

**Table 4:** Twelve modes and their associated modal and system parameters.

	Ω_1_	Ω_2_	Ω_3_	Ω_4_	Ω_5_	Ω_6_	Ω_7_	Ω_8_	Ω_9_	Ω_10_	Ω_11_	Ω_12_
**Average *f*** (Hz)	4.28	7.61	13.15	21.98	56.70	71.59	92.71	133.73	208.79	322.24	494.74	664.02
**Minimum *f*** (Hz)	3.00	4.70	10.00	18.75	51.18	62.90	82.40	113.67	158.44	263.10	411.67	604.17
**Maximum *f*** (Hz)	5.08	11.00	16.70	26.50	66.70	84.00	103.50	168.50	270.67	404.63	536.25	741.00
**Standard deviation *f*** (Hz)	0.42	0.34	0.71	0.71	1.69	1.91	4.66	4.34	6.40	8.99	11.94	16.08
**Relative power** (dB)	−12.5	−13.8	−12.4	−11.7	−0.3	−0.8	−1.4	−5.3	−10.1	−14.9	−17.8	−18.8
**Amplitude cross-spectrum** ((m/s^2^)^2^/Hz)	3.71 × 10^−3^	2.88 × 10^−3^	3.48 × 10^−3^	1.92 × 10^−3^	3.44 × 10^−2^	2.53 × 10^−2^	1.85 × 10^−2^	1.04 × 10^−2^	4.11 × 10^−3^	1.23 × 10^−3^	7.31 × 10^−4^	5.31 × 10^−4^
**Phase** (Radian)	0.87π	0.65π	0.75π	0.92π	0.87π	0.82π	0.73π	0.69π	0.59π	0.64π	0.60π	0.53π
**Mode**	OOP	Coupled	OOP	OOP	OOP	OOP	Coupled	Coupled	Coupled	Coupled	Coupled	Coupled
**Coherence**	0.93	0.90	0.89	0.94	0.99	0.99	0.98	0.99	0.97	0.96	0.95	0.95
**Damping ratio**	0.22	0.17	0.17	0.14	0.19	0.21	0.18	0.14	0.13	0.12	0.11	0.09
**Number of samples**	33	35	36	7	32	21	16	21	22	26	26	30

**Table 5:** Median values of the body components for observation and no observation groups of $\Omega_{7}$, $\Omega_{8}$, and $\Omega_{10}$ with significance p<0.05 (unless indicated otherwise) based on the Wilcoxon rank sum test.

	GROUP	*h*	*t_L_*	*t_U_*	*l*	*w*	*s_L_*	*s_F_*
**Ω_7_**	*O*	-	-	-	-	66.3 *	7.975 **	-
	*N*	-	-	-	-	72.4 *	8.77 **	-
**Ω_8_**	*O*	173.6	43.5	51.1	95	**75.2**	**8.92**	2.8 ^
	*N*	169.9	42	49.1	92	**61.8**	**7.2**	1.8 ^
**Ω_10_**	*O*	-	-	-	-	64	7.975	-
	*N*	-	-	-	-	73.85	8.875	-

(bold) *p* < 0.01; * p~0.06  for weight and ** p~0.08 for segmental lean leg mass for groups of $\Omega_{7}$; ^ p~0.06 for segmental fat leg mass for groups of $\Omega_{8}$.

**Table 6:** Models for modes 6–11 and their coefficients of variables and constants.

Modes	Equation	$\mathrm{Constant}\;n_{0}$			Coefficient		
		Value	SE	p	Value	SE	p
					$n_{L_{F}}$		
$F_{6}$	$n_{0}+n_{L_{f}}\cdot L_{f}$	9.12	1.30	$\sim 10^{-6}$	−1.23	0.33	0.001
$F_{7}$		10.08	1.49	$\sim 10^{-5}$	−1.41	0.38	0.002
$F_{11}$		8.72	1.06	$\sim 10^{-8}$	−0.64	0.27	0.026
					$n_{S_{L}}$		
$F_{8}$	$n_{0}+n_{S_{L}}\cdot S_{L}$	5.70	0.32	$\sim 10^{-13}$	−0.37	0.14	0.019
					$n_{S_{F}}$		
$F_{9}$	$n_{0}+n_{S_{F}}\cdot S_{F}$	5.48	0.07	$\sim 10^{-26}$	−0.17	0.07	0.024
$F_{10}$		5.86	0.04	$\sim 10^{-36}$	−0.10	0.04	0.030
